# PDE1 or PDE5 inhibition augments NO‐dependent hypoxic constriction of porcine coronary artery via elevating inosine 3′,5′‐cyclic monophosphate level

**DOI:** 10.1111/jcmm.16078

**Published:** 2020-11-09

**Authors:** Yan Nan, Xueqin Zeng, Zhiyi Jin, Na Li, Zhengju Chen, Jiantong Chen, Dezhong Wang, Yang Wang, Zhenlang Lin, Lei Ying

**Affiliations:** ^1^ Department of Neonatology The Second Affiliated Hospital and Yuying Children's Hospital Wenzhou Medical University Wenzhou China; ^2^ Department of Pathophysiology School of Basic Medical Sciences Wenzhou Medical University Wenzhou China; ^3^ Department of Pathology Wenzhou Central Hospital Wenzhou China; ^4^ Department of Physiology and Pathophysiology Peking University Health Science Center Beijing China; ^5^ Institute of Life Sciences Wenzhou University Wenzhou China

**Keywords:** coronary artery, hypoxic constriction, inosine 3′,5′‐cyclic monophosphate, nitric oxide, phosphodiesterase

## Abstract

Hypoxic coronary vasospasm may lead to myocardial ischaemia and cardiac dysfunction. Inosine 3′,5′‐cyclic monophosphate (cIMP) is a putative second messenger to mediate this pathological process. Nevertheless, it remains unclear as to whether levels of cIMP can be regulated in living tissue such as coronary artery and if so, what is the consequence of this regulation on hypoxia‐induced vasoconstriction. In the present study, we found that cIMP was a key determinant of hypoxia‐induced constriction but not that of the subsequent relaxation response in porcine coronary arteries. Subsequently, coronary arteries were treated with various phosphodiesterase (PDE) inhibitors to identify PDE types that are capable of regulating cIMP levels. We found that inhibition of PDE1 and PDE5 substantially elevated cIMP content in endothelium‐denuded coronary artery supplemented with exogenous purified cIMP. However, cGMP levels were far lower than their levels in intact coronary arteries and lower than cIMP levels measured in endothelium‐denuded coronary arteries supplemented with exogenous cIMP. The increased cIMP levels induced by PDE1 or PDE5 inhibition further led to augmented hypoxic constriction without apparently affecting the relaxation response. In intact coronary artery, PDE1 or PDE5 inhibition up‐regulated cIMP levels under hypoxic condition. Concomitantly, cGMP level increased to a comparable level. Nevertheless, the hypoxia‐mediated constriction was enhanced in this situation that was largely compromised by an even stronger inhibition of PDEs. Taken together, these data suggest that cIMP levels in coronary arteries are regulated by PDE1 and PDE5, whose inhibition at a certain level leads to increased cIMP content and enhanced hypoxic constriction.

## 1 INTRODUCTION

Coronary artery spasm (CAS) refers to transient coronary artery constriction that may lead to incomplete or complete blood vessel closure and myocardial ischaemia.^1^, ^2^ Clinically, CAS may elicit angina, arrhythmia, myocardial infarction and even death. The pathogenesis of CAS includes dysfunction of vascular endothelium, hypersensitivity of vascular smooth cell, oxidative stress, chronic inflammation and increased autonomic nerve activity.^1^, ^2^ In addition, hypoxia‐induced constriction of coronary arteries (also known as hypoxic coronary vasospasm) is a potential candidate for the mechanism of CAS. Impairment or dysfunction of endothelium remarkably potentiates the acute hypoxic constriction of canine coronary artery, substantially decreasing the blood supply to cardiac muscle.^3^ As in the case for example in sleep apnoea, the hypoxic constriction that is repeated consistently even after several episodes of exposure to hypoxia could be a vital risking factor in patients (especially for those with a previous history of coronary disease) exposed to repeated episodes of reduced oxygen in the blood.^4^, ^5^, ^6^

Hypoxic constriction of coronary arteries is independent of vasoconstrictors ^3^, ^7^, ^8^, ^9^; however, it entails endothelial released nitric oxide (NO) (considered a classical vasodilator).^7^, ^8^, ^9^, ^10^, ^11^, ^12^ The removal of endothelium substantially blunts this hypoxic response, while the addition of NO restores this reaction.^9^, ^10^ Apart from NO, the hypoxic response relies on the activation of its downstream signal, soluble guanylyl cyclase (sGC).^9^, ^13^ Normally, NO activates sGC to elevate intracellular guanosine 3′,5′‐cyclic monophosphate (cGMP) levels that further induce vasodilation.^13^, ^14^ However, the reported evidence does not support the notion that this hypoxic response requires cGMP.^9^, ^10^ Although cGMP is a well‐established second messenger that is formed by catalysation of sGC,^13^, ^15^ it has been postulated that there may be other cGMP‐like molecules produced via sGC activation with signal‐transducing functions.^16^, ^17^, ^18^ This hypothesis is supported by findings of a study using purified recombinant human sGC in which the enzyme catalysed the formation of cGMP and that of adenosine 3′,5′‐cyclic monophosphate (cAMP), inosine 3′,5′‐cyclic monophosphate (cIMP), cytidine 3′,5′‐cyclic monophosphate, uridine 3′,5′‐cyclic monophosphate and xanthosine 3′,5′‐cyclic monophosphate.^19^ Except for cGMP, the maximal rate formation of cIMP is substantially greater than that of other nucleosides.^19^, ^20^ In a previous study, we found that the activation of the NO‐sGC axis led to increased levels of cIMP (proposed as a novel second messenger ^21^), that further activated Rho kinase, inhibited myosin light chain phosphatase activation and induced constriction in porcine coronary artery.^9^

Studies using purified recombinant human phosphodiesterases (PDEs) and 3′,5′‐cyclic nucleotide salts showed that cIMP is hydrolysed by a number of types of PDEs as in the case of cGMP.^22^, ^23^ However, it remains unclear as to whether this regulating mode is the same in living tissues, and if so, what are the consequences for hypoxic constriction. To address this question, we investigated the effects of various PDE inhibitors on cIMP levels and hypoxia‐induced constriction in porcine coronary arteries with or without endothelium.

## 2 MATERIALS AND METHODS

### 2.1 Tissue preparations

Fresh porcine hearts were collected from a local slaughter house and were submerged in oxygenated (aerated with 95% O_2_ plus 5% CO_2_ for at least 30 minutes) ice‐cold modified Krebs‐Ringer bicarbonate solution (composition (mM): NaCl, 118.3; KCl, 4.7; CaCl_2_, 2.5; MgSO_4_, 1.2; KH_2_PO_4_, 1.2; NaHCO_3_, 25.0; glucose, 11.1). Left anterior descending coronary arteries of the hearts (outside diameter = 2‐3 mm) were immediately dissected in the ice‐cold Krebs‐Ringer solution. In some experiments, the tips of a watchman's forceps were used to mechanically remove endothelium of coronary artery by gently rubbing the luminal surface the on a tissue paper soaked in Krebs solution. Lack of relaxation to bradykinin was used as a standard to confirm the removal of endothelium.

### 2.2 Vessel tension studies

Coronary artery rings (length: 3‐5 mm) were suspended in organ chambers filled with 10 mL of Krebs solution maintained at 37 ± 1°C and aerated with 95% O_2_ and 5% CO_2_ (pH = 7.4). Each ring was suspended by two stirrups passing through its lumen; one stirrup was anchored to the bottom of organ chamber and the other connected to a strain gauge (Power‐Lab/8sp, ADI Instrument, Australia) for measuring isometric force.

At the beginning, coronary artery rings were stretched to its optimal resting tension (~2.5 g) by stepwise stretching followed by an hour equilibration. Then, the rings were constricted with KCl (100 mmol/L) twice until the active contraction reached a plateau for each time. Subsequently, the rings were incubated with various inhibitors (including indomethacin (10^−5^ mol/L), nitro‐l‐arginine (NLA) (10^−4^ mol/L), 1H‐[1,2,4]oxadiazolo[4,3‐a]quinoxalin‐1‐one (ODQ) (3 × 10^−5^ mol/L) (dissolved in DMSO, final concentration < 0.01%) and 8‐Br‐cGMP (10^−5^ or 3 × 10^−5^ mol/L) and/or DETA NONOate (a NO donor) (3 × 10^−6^ mol/L) for at least 30 mins to allow cell permeation and full inhibition of the target (remained throughout the experiment). Before exposure to hypoxia, the rings were contracted with U‐46619 (a thromboxane A2 mimics) (3 × 10^−7^ mol/L) to reach a plateau and incubated with PDE inhibitors (including 3‐isobutyl‐1‐methylxanthine (IBMX) (10^−5^ mol/L), 8‐methoxymethyl‐IBMX (4 × 10^−5^ mol/L), zaprinast (10^−5^ mol/L), milrinone (5 × 10^−6^ mol/L) and rolipram (5 × 10^−6^ mol/L), dissolved in DMSO or ethanol (8‐methoxymethyl‐IBMX), final concentration < 0.01%). Ten minutes of hypoxic exposure to the rings were performed using 95% N_2_ and 5% CO_2_. Indomethacin or NLA was added in experiments to eliminate the possible involvement of endogenous prostanoids or NO, respectively. Indomethacin was dissolved in distilled water with equimolar Na_2_CO_3_. DETA NONOate was prepared by dissolving in distilled water containing 0.01 mol/L NaOH. The concentration of Na_2_CO_3_ or NaOH had no significant effect on the pH of the solution.

Endothelium‐denuded coronary artery rings were incubated with indomethacin (10^−5^ mol/L) and NLA (10^−4^ mol/L) for at least 30 minutes. Then, the rings were contracted with U‐46619 (6 × 10^−8^ mol/L), treated with cIMP plus corresponding PDE inhibitor (from 5 × 10^−6^ mol/L to 4 × 10^−5^ mol/L) or solvent control and exposed to hypoxia for 10 minutes.

### 2.3 In vitro incubation study

For the cIMP incubation study, endothelium‐denuded coronary artery rings were suspended in tubes filled with 5 mL of Krebs solution, maintained at 37 ± 1°C and aerated with 95% O_2_ and 5% CO_2_ (pH = 7.4). The rings were equilibrated for 30 minutes and treated with indomethacin (10^−5^ mol/L) and NLA (10^−4^ mol/L) for 30 minutes. Then, the rings were incubated with cIMP (3 × 10^−5^ mol/L) for another 30 minutes, snap‐frozen in liquid nitrogen and subjected to the detection and quantification of cIMP levels. During this process, the PDE inhibitors (all at 2 × 10^−5^ mol/L) were added 5 minutes after cIMP treatment and remained throughout the experiment.

To measure cIMP levels in intact coronary artery rings under hypoxia, coronary artery rings suspended in tubes with 5 mL of Krebs solution were equilibrated for 30 minutes and treated with indomethacin (10^−5^ mol/L) for 30 minutes. Then, the rings were incubated with U‐46619 (3 × 10^−7^ mol/L) for 30 minutes and exposed to hypoxia for 3 minutes (for hypoxic groups). After all treatments, the rings were snap‐frozen in liquid nitrogen and subjected to the detection and quantification of cIMP levels. During this process, the PDE inhibitors (all at 2 × 10^−5^ mol/L) were added 5 minutes after U‐46619 treatment and remained throughout the experiment.

### 2.4 UPLC/MS/MS method for the quantification of cIMP and cGMP

After incubation, the frozen coronary artery tissue (40‐100 mg) was homogenized and dissolved in 100% methanol containing 10 ng/mL tenofovir as an internal standard (IS) and centrifuged at 12 000 *g* for 20 minutes (4°C). The supernatants were collected and dried using Termovap Sample Concentrator. Subsequently, the powder was dissolved in 120 μL pure water and filtered with a 0.22 μm filter for ultra‐performance liquid chromatography (UPLC)‐MS/MS analysis.

The detection and quantification of cIMP and cGMP levels in porcine coronary arteries were performed using an ACQUITYI‐Class UPLC system equipped with a XEVO TQS‐micro triple quadrupole mass spectrometry (Waters Corp., Milford, MA, USA).^9^, ^24^ After the injection of 2 μL volume, analysts were separated using an ACQUITY UPLC^®^ BEH C18 (2.1 × 50 mm, 1.7 μm) column at 40°C. The gradient mobile phases consisted of solvent A (Milli‐Q pure water containing 0.01% formic acid and 0.05% ammonia) and solvent B (acetonitrile containing 0.01% formic acid). The initial gradient containing 97% solvent A and 3% solvent B was maintained for 1 minute, and the fraction of solvent B was then raised to 15% in 1 minute, held for 2 minutes, restored to starting conditions in 1 minute and held for 1 minute. The flow rate was 0.4 mL/min.

Mass detection was performed on the micro triple quadrupole mass spectrometry with ESI source operated in positive ion mode. The multiple selected ion monitoring transitions were detected with a 54‐ms dwell time. The optimized mass spectrometer parameters were set as follows: capillary voltage, 3 kV; and desolvation temperature, 450°C. The optimal collision energies of cIMP, cGMP and IS were 18, 20 and 20 V, respectively. The cone voltage was set at 25 V for cGMP and IS and 20 V for cIMP. The ion transitions of MRM were m/z 331 → 137, m/z 346 → 152 and m/z 288 → 176 for cIMP, cGMP and IS, respectively. Data were acquired and analysed with Masslynx 4.1 software (Waters Corp.).

### 2.5 Statistical analyses

Data were expressed as mean ± SEM. Hypoxia‐induced contractions were expressed as percentage of the reference contraction to U‐46619 (taken as 100%) in coronary arteries. Student's unpaired *t* tests were used to compare two groups. In the comparison of mean values of more than two groups, one‐way analysis of variance (ANOVA) test with Tukey's multiple comparisons test was used. Two‐way ANOVA test with Sidak's multiple comparisons test was used to compare two or more than two groups when the time course of treatment was investigated. *P*‐values (two tailed) less than .05 were considered statistically significant. N represents the number replicated in corresponding experiment.

## 3 RESULTS

### 3.1 NO‐sGC‐cIMP signalling axis determines hypoxic vasoconstriction of porcine coronary artery

**Figure 1:**
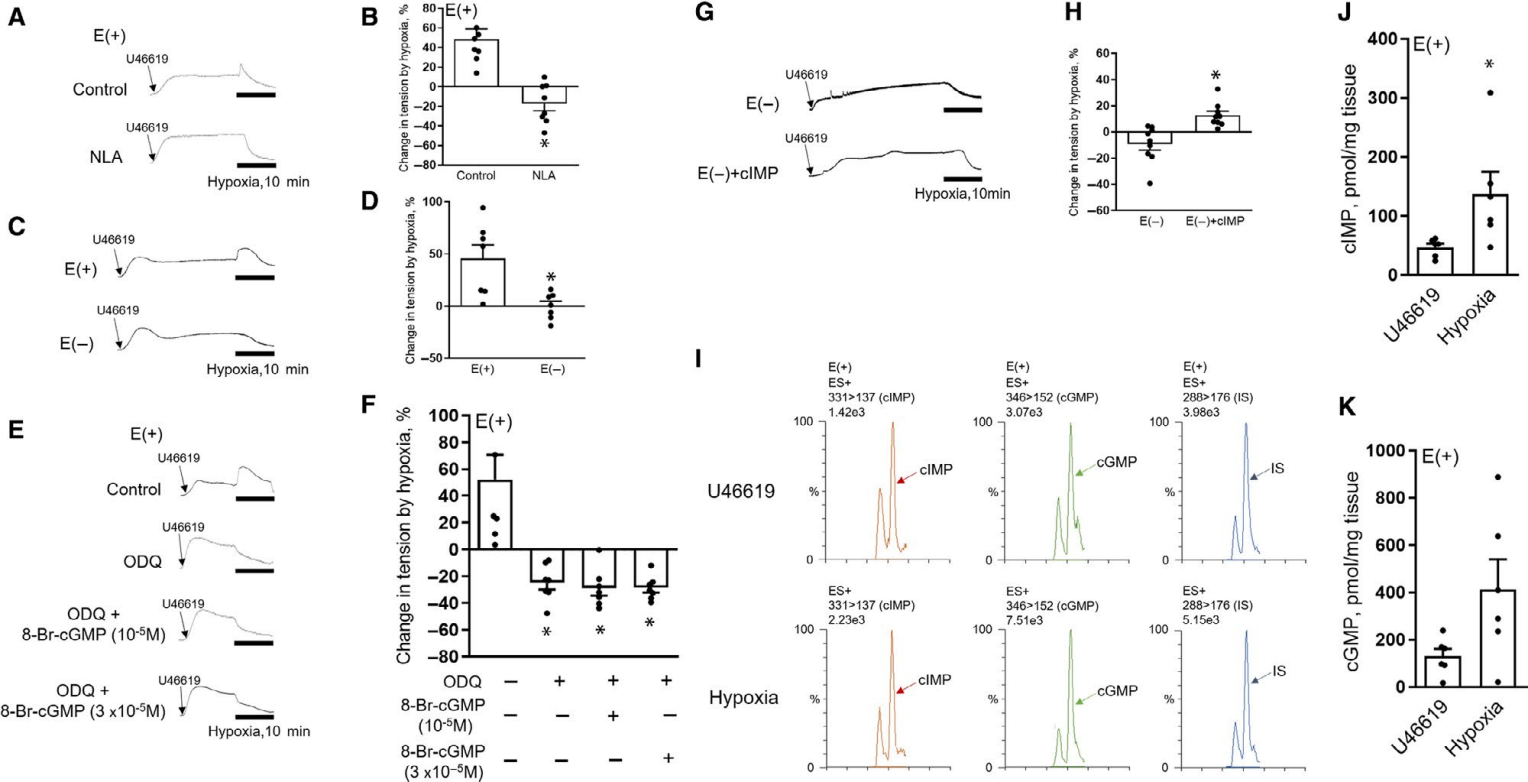
Hypoxia‐induced vasoconstriction of porcine coronary artery entails the activation of NO‐sGC‐cIMP signalling axis. A, B, Original traces (A) and summaries (B) of hypoxic responses of porcine coronary arteries pre‐treated with indomethacin (10^−5^ mol/L) plus NLA (10^−4^ mol/L) or solvent control for at least 30 min and contracted with U‐46619 (3 × 10^−7^ mol/L) (n = 8). C, D, Original traces (C) and summaries (D) of hypoxic responses of porcine coronary arteries (with or without endothelium) pre‐treated with indomethacin (10^−5^ mol/L) for at least 30 min and contracted with U‐46619 (3 × 10^−7^ mol/L) (n = 7). E, F, Original traces (E) and summaries (F) of hypoxic responses of porcine coronary arteries pre‐treated with indomethacin (10^−5^ mol/L) and/or ODQ (3 × 10^−5^ mol/L) for at least 30 min, incubated with 8‐Br‐cGMP (10^−5^or 3 × 10^−5^ mol/L) or solvent control and contracted with U‐46619 (3 × 10^−7^ mol/L) (n = 7). G, H, Original traces (G) and summaries (H) of hypoxic responses of endothelium‐denuded porcine coronary arteries pre‐treated with indomethacin for at least 30 min, contracted with U‐46619 (6 × 10^−8^ mol/L) and incubated with cIMP (10^−4^ mol/L) (n = 9). I‐K, Original ultra‐performance liquid chromatography‐MS/MS traces (I) and summaries of the effects of hypoxia on levels of cIMP (J) and cGMP (K) in porcine coronary arteries pre‐treated with indomethacin (10^−5^ mol/L) for at least 30 min, incubated with U‐46619 (3 × 10^−7^ mol/L) for 30 min and exposed to hypoxia for 3 min (n = 6). Tenofovir (10 ng/mL) was served as an internal standard (IS). E (+), with endothelium. E (−), without endothelium. All data are presented as mean ± SEM. *P < .05 vs Control, E (+), E (−), or U‐46619. Statistical comparisons in B, D, H, J and K are unpaired two‐tailed Student's*t*tests. Statistical comparison in F is one‐way ANOVA test with Tukey's multiple comparisons test

As previously reported, hypoxia (95% N_2_ plus 5% CO_2_) induced an acute and transient constricting response in porcine coronary artery aerated with 95% O_2_ plus 5% CO_2_ ^9^, ^10^ (Figure 1A‐F and Figure S1). This response was substantially abrogated by a nitric oxide synthase inhibitor, nitro‐l‐arginine (NLA) (10^−4^ mol/L) (Figure 1A,B), the removal of endothelium (Figure 1C,D) and an inhibitor of sGC (ODQ) (3 × 10^−5^ mol/L) (Figure 1E,F).

To further explore the roles of potential sGC downstream targets (including cGMP and cIMP) in hypoxia‐induced constriction of coronary artery, exogenous supply of cGMP or cIMP was used in the subsequent experiment. We found that elevating of cGMP level by its exogenous supplement (8‐Br‐cGMP, at 10^−5^ or 3 × 10^−5^ mol/L) failed to initiate the hypoxic constriction blocked by ODQ (3 × 10^−5^ mol/L) (Figure 1E,F). However, hypoxia restored the constricting response in the presence of cIMP (10^−4^ mol/L) in endothelium‐denuded coronary artery (Figure 1G,H). Consistent with this finding, the UPLC/MS/MS analyses showed that 3 minutes of hypoxia induced a robust increase of cIMP levels in coronary arteries (Figure 1I,J and Figure S2A,B). Although the reduction of cGMP level is another possible mechanism that may contribute to hypoxic constriction, there was no significant decrease but rather a tendency of increase of its levels in hypoxic conditions (Figure 1I,K and Figure S2A,B). Taken together, these data suggest that hypoxic constriction of porcine coronary artery involves the activation of NO‐sGC signalling and downstream elevation of cIMP levels.

### 3.2 Inhibition of PDE1 or PDE5 elevates cIMP levels in endothelium‐denuded porcine coronary artery treated with exogenous cIMP

**Figure 2:**
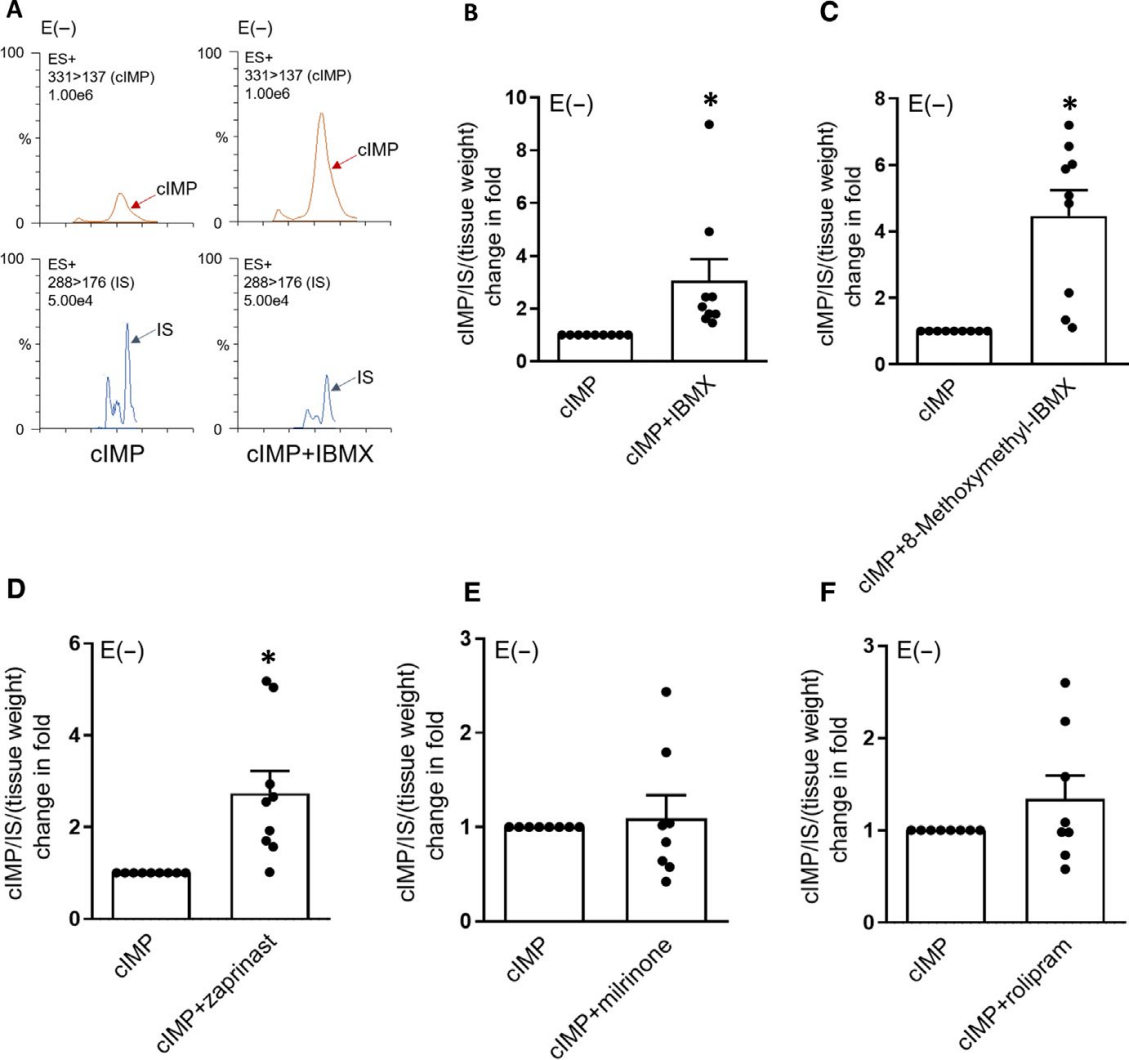
CIMP levels are up‐regulated by the inhibition of PDE1 or PDE5 in endothelium‐denuded porcine coronary artery treated with exogenous cIMP. A‐F, Original ultra‐performance liquid chromatography‐MS/MS traces (A) and summaries of fold change of cIMP in endothelium‐denuded porcine coronary arteries pre‐treated with indomethacin (10^−5^ mol/L) and NLA (10^−4^ mol/L) and incubated with exogenous cIMP (3 × 10^−5^ mol/L) plus IBMX (B), 8‐methoxymethyl‐IBMX (C), zaprinast (D), milrinone (E), rolipram (F) (all at 2 × 10^−5^ mol/L) or solvent control for 30 min (n = 8‐9). Tenofovir (10 ng/mL) was served as an internal standard (IS). E (−), without endothelium. All data are presented as mean ± SEM. *P < .05 vs cIMP. Statistical comparisons in B‐F are unpaired two‐tailed Student's*t*tests

Although studies using purified PDEs and 3′,5′‐cyclic nucleotides has shown that cIMP level is hydrolysed by several types of PDE,^22^, ^23^ it remains uncertain whether this regulating mode is applicable in the living tissues such as coronary artery. We found that the 25 minutes treatment of IBMX (a broad‐spectrum PDE inhibitor ^25^, ^26^) (2 × 10^−5^ mol/L) remarkably increased the cIMP levels in endothelium‐denuded porcine coronary artery treated with high levels of purified cIMP (3 × 10^−5^ mol/L) (Figure 2A,B) (in the presence of NLA, at 10^−4^ mol/L). This indicates that the degradation velocity of cIMP is delayed by broad PDE inhibition.

**Figure 3:**
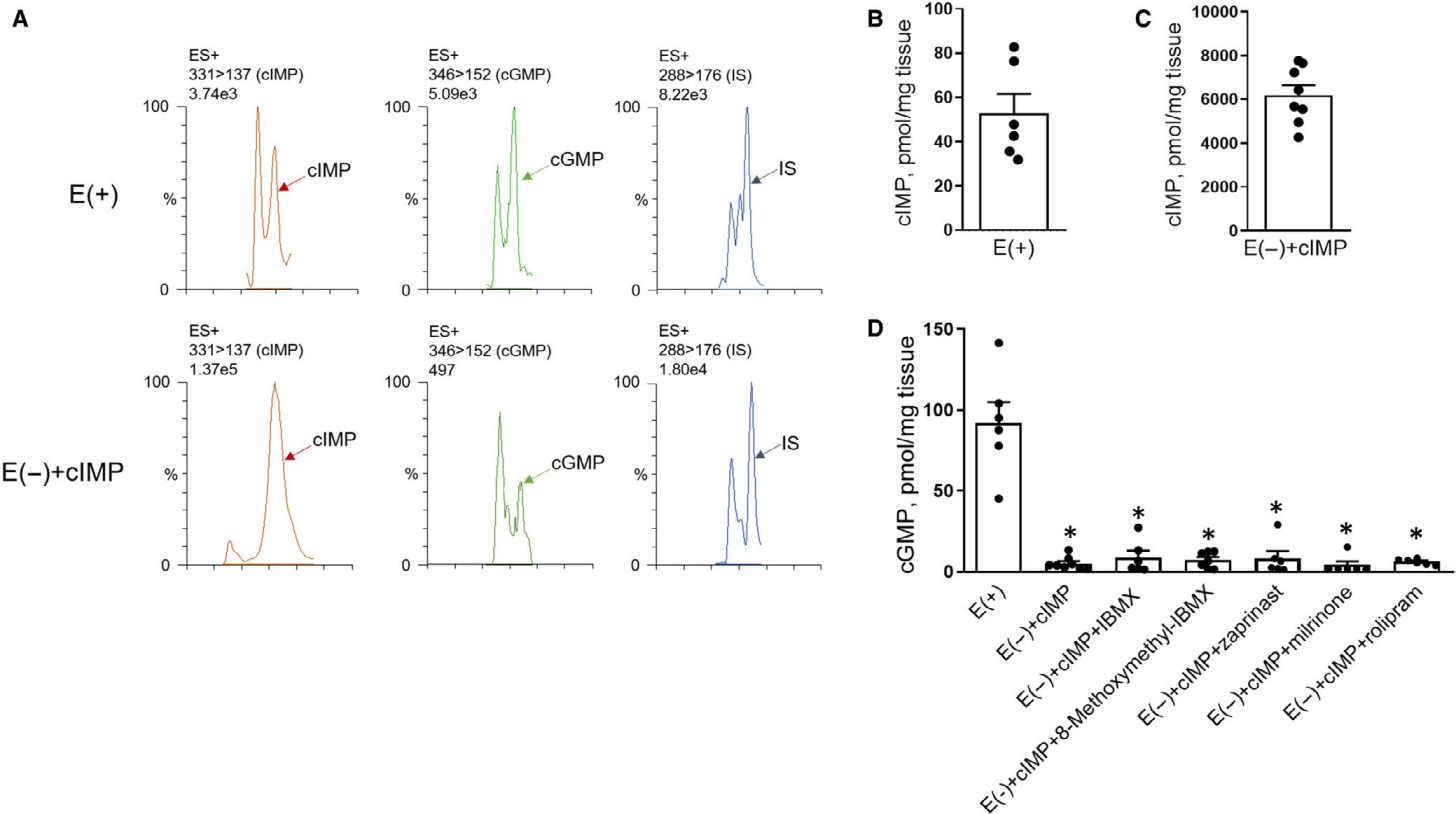
CIMP and cGMP levels in porcine coronary arteries with or without endothelium. A‐D, Original ultra‐performance liquid chromatography‐MS/MS traces (A) and summaries of levels of cIMP (B and C) and cGMP (D) in intact porcine coronary arteries or endothelium‐denuded coronary arteries pre‐treated with indomethacin (10^−5^ mol/L) and NLA (10^−4^ mol/L) and incubated with exogenous cIMP (3 × 10^−5^ mol/L) plus IBMX, 8‐methoxymethyl‐IBMX, zaprinast, milrinone, rolipram (all at 2 × 10^−5^ mol/L) or solvent control for 30 min (all at 2 × 10^−5^ mol/L) (n = 6‐8). Tenofovir (10 ng/mL) was served as an internal standard (IS). E (+), with endothelium. E (−), without endothelium. All data are presented as mean ± SEM. *P < .05 vs E (+). Statistical comparison in D is one‐way ANOVA test with Tukey's multiple comparisons test

To determine the subtypes of PDE that regulate cIMP levels in coronary artery, several selective PDE inhibitors were used in the subsequent experiments (according to published studies^22^, ^23^). We found that 8‐methoxymethyl‐IBMX (a PDE1 inhibitor^27^, ^28^) and zaprinast (a PDE5 inhibitor^29^, ^30^) (all at 2 × 10^−5^ mol/L) elevated cIMP levels in coronary arteries (without endothelium and with the existence of exogenous cIMP and NLA) compared to levels in the control group (Figure 2C,D). However, milrinone (a PDE3 inhibitor^31^, ^32^) and rolipram (a PDE4 inhibitor^33^, ^34^) (all at 2 × 10^−5^ mol/L) had no apparent effect on cIMP level compared to the control (Figure 2E,F). Because of the removal of endothelium and treatment with NLA, cGMP levels in all groups were far lower than their levels and cIMP levels in intact coronary arteries (with endothelium and without any treatment) (Figure 3A,B,D) and lower than cIMP levels measured in endothelium‐denuded coronary artery (supplemented with exogenous cIMP) (Figure 3A,C). Taken together, these data suggest that cIMP levels in coronary artery are regulated by PDE1 or PDE5, whose inhibition in turn induces significant increases in intracellular cIMP levels.

### 3.3 Inhibition of PDE1 or PDE5 augments cIMP‐mediated hypoxic vasoconstriction in endothelium‐denuded porcine coronary artery

**Figure 4:**
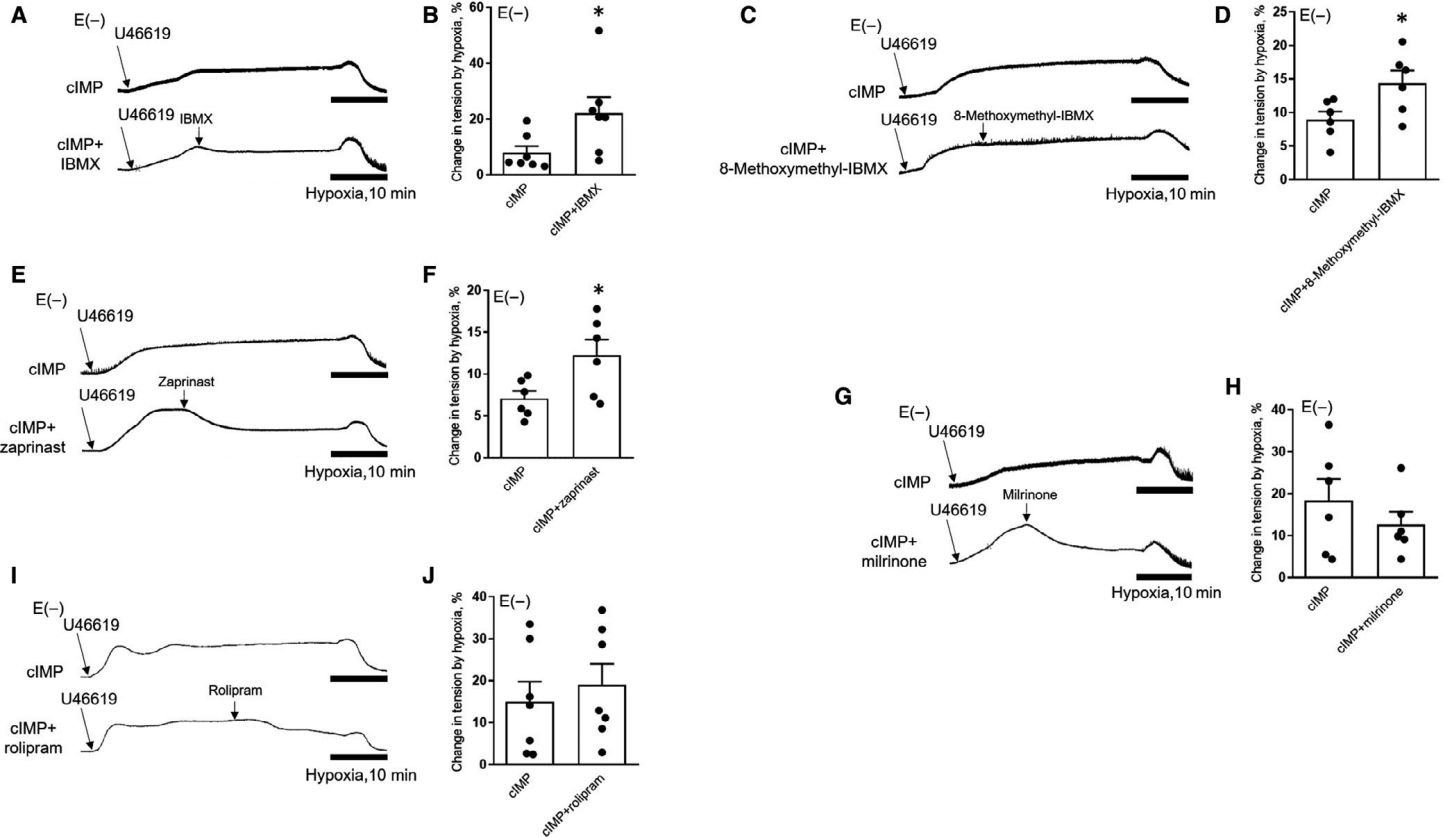
CIMP‐mediated hypoxic vasoconstriction is potentiated by the inhibition of PDE1 or PDE5 in endothelium‐denuded porcine coronary artery. A‐J, Original traces (A, C, E, G and I) and summaries (B, D, F, H and J) of hypoxic responses of endothelium‐denuded coronary arteries pre‐treated with indomethacin (10^−5^ mol/L) and NLA (10^−4^ mol/L) for at least 30 min, contracted with U‐46619 (6 × 10^−8^ mol/L) and incubated with exogenous cIMP (10^−4^ mol/L) plus IBMX (3 × 10^−5^ mol/L) (A and B), 8‐methoxymethyl‐IBMX (4 × 10^−5^ mol/L) (C and D), zaprinast (10^−5^ mol/L) (E and F), milrinone (5 × 10^−6^ mol/L) (G and H), rolipram (5 × 10^−6^ mol/L) (I and J) or solvent control (n = 6‐7). E (−), without endothelium. All data are presented as mean ± SEM. *P < .05 vs cIMP. Statistical comparisons in B, D, F, H and J are unpaired two‐tailed Student's*t*tests

We previously showed that the strength of hypoxic constriction in porcine coronary artery is determined by relative quantities of cIMP in the tissue.^9^ Given that the inhibition of PDEs including PDE1 and PDE5 increase cIMP levels in endothelium‐denuded coronary artery supplemented with exogenous cIMP, we next determined whether the up‐regulation of cIMP levels would further enhance hypoxic constriction. As expected, IBMX (3 × 10^−5^ mol/L) augmented hypoxia‐induced constriction of porcine coronary arteries (without endothelium and pre‐treated with NLA (10^−4^ mol/L)) with the addition of exogenous cIMP (10^−4^ mol/L) (Figure 4A,B). More specifically, this potentiated hypoxic constriction involves the inhibited functions of PDE1 (using 8‐methoxymethyl‐IBMX) (4 × 10^−5^ mol/L) (Figure 4C,D) or PDE5 (using zaprinast) (Figure 4E,F) (10^−5^ mol/L). Despite the fact that cIMP‐mediated hypoxic constriction was preserved in coronary arteries treated with milrinone (5 × 10^−6^ mol/L) or rolipram (5 × 10^−6^ mol/L), there was no significant difference compared to the tension induced by cIMP alone (Figure 4G‐J). Taken together, these data suggest that increased cIMP levels in coronary artery induced by PDE1 or PDE5 inhibition enhance cIMP‐mediated hypoxic constriction of porcine coronary arteries without endothelium.

### 3.4 Inhibition of PDE1 or PDE5 potentiates cIMP levels and associated vasoconstriction in intact porcine coronary arteries under hypoxic conditions

**Figure 5:**
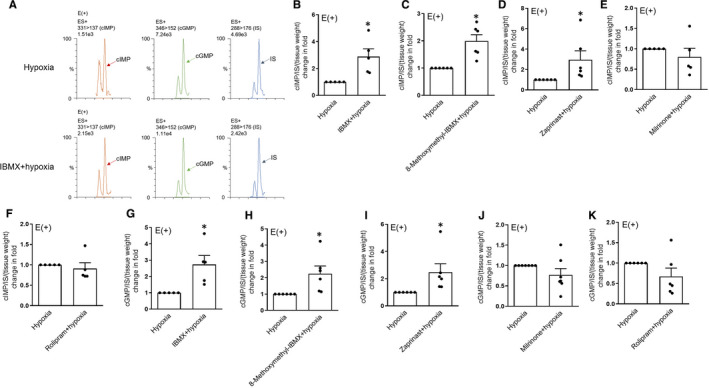
CIMP and cGMP levels are up‐regulated by the inhibition of PDE1 or PDE5 in intact porcine coronary arteries under hypoxia. A‐K, Original ultra‐performance liquid chromatography‐MS/MS traces (A) and summaries of fold change of cIMP (B‐F) and cGMP (G‐K) in intact porcine coronary arteries pre‐treated with indomethacin (10^−5^ mol/L), incubated with U‐46619 (3 × 10^−7^ mol/L) plus IBMX (A, B and G), 8‐methoxymethyl‐IBMX (C and H), zaprinast (D and I), milrinone (E and J), rolipram (F and K) (all at 2 × 10^−5^ mol/L) or solvent control for 30 min and exposed to hypoxia for 3 min (n = 5‐7). Tenofovir (10 ng/mL) was served as an internal standard (IS). E (+), with endothelium. All data are presented as mean ± SEM. *P < .05 vs hypoxia. Statistical comparisons in B‐K are unpaired two‐tailed Student's*t*tests

To explore the effect of PDE inhibition on cIMP levels and vessel tension under hypoxic condition in intact porcine coronary artery (with endothelium), these PDE inhibitors were applied prior to exposure to hypoxia. In these conditions, IBMX (2 × 10^−5^ mol/L) induced a robust elevation of cIMP levels compared to pure hypoxia treatment (Figure 5A,B). Consistent with this finding, the increase of cIMP content also occurred following 8‐methoxymethyl‐IBMX (Figure 5C) or zaprinast treatment (Figure 5D) (all at 2 × 10^−5^ mol/L). Inhibition of PDE3 or PDE4 by milrinone or rolipram (all at 2 × 10^−5^ mol/L) had little effect on cIMP levels (Figure 5E,F). Concomitant with changes in cIMP levels, we found that levels of cGMP were elevated by IBMX, 8‐methoxymethyl‐IBMX and zaprinast but not by milrinone or rolipram (all at 2 × 10^−5^ mol/L) (Figure 5A,G‐K).

**Figure 6:**
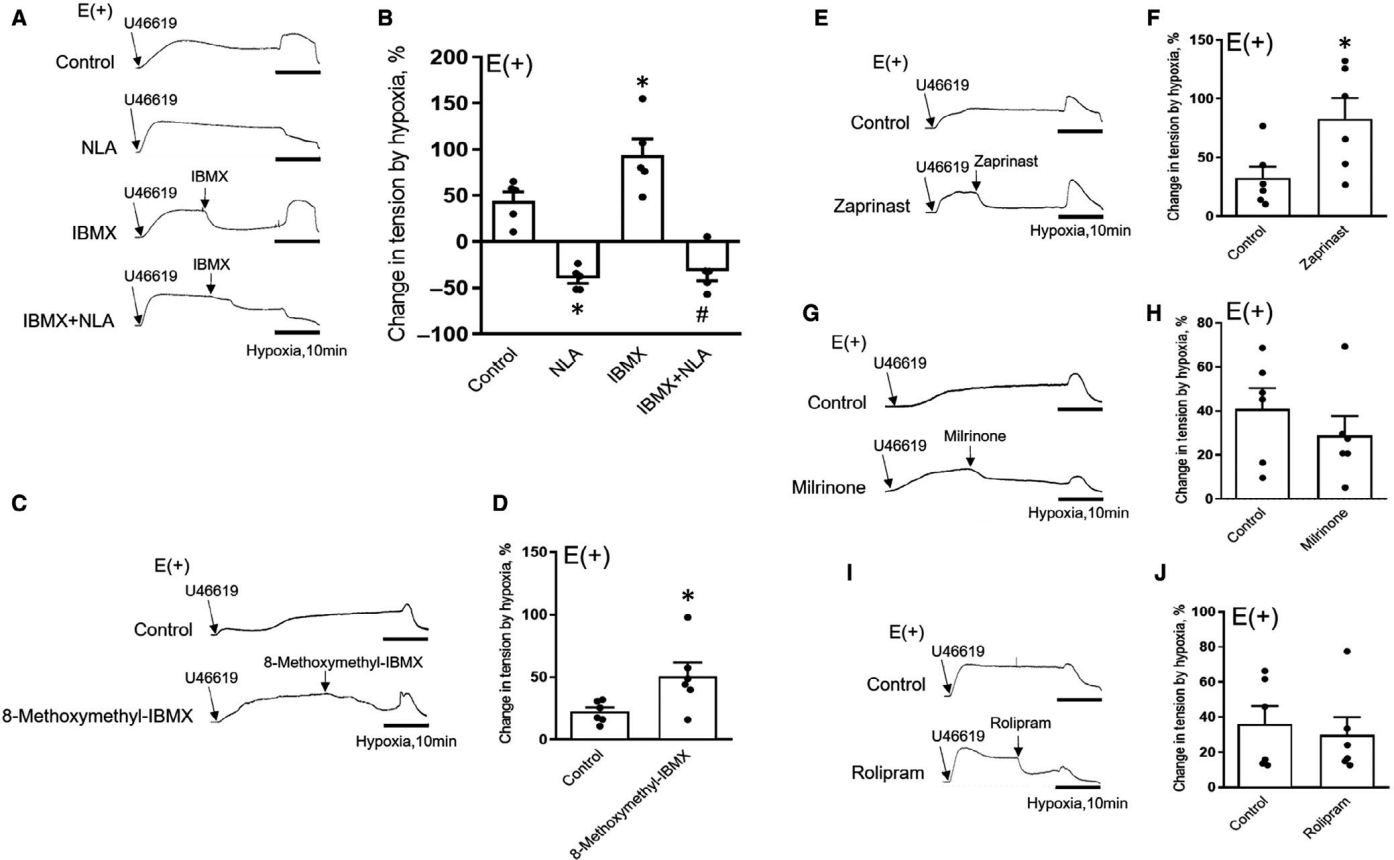
Hypoxic vasoconstriction of intact porcine coronary artery is enhanced by the inhibition of PDE1 or PDE5. A‐J, Original traces (A, C, E, G and I) and summaries (B, D, F, H and J) of hypoxic responses of intact coronary arteries pre‐treated with indomethacin (10^−5^ mol/L) and/or NLA (10^−4^ mol/L) for at least 30 min, contracted with U‐46619 (3 × 10^−7^ mol/L) and incubated with IBMX (10^−5^ mol/L) (A and B), 8‐methoxymethyl‐IBMX (4 × 10^−5^ mol/L) (C and D), zaprinast (10^−5^ mol/L) (E and F), milrinone (5 × 10^−6^ mol/L) (G and H), rolipram (5 × 10^−6^ mol/L) (I and J) or solvent control (n = 5‐6). E (+), with endothelium. All data are presented as mean ± SEM. *P < .05 vs control;^#^ *P* < .05 vs IBMX. Statistical comparison in B is one‐way ANOVA test with Tukey's multiple comparisons test. Statistical comparisons in D, F, H and J are unpaired two‐tailed Student's*t*tests.

Therefore, we further explored whether inhibition of PDE1 and PDE5 would induce enhanced constricting responses in intact coronary arteries exposed to hypoxia. Unlike endothelium‐denuded coronary arteries exposed to NLA, which completely abolished intramuscular cGMP levels, cGMP was at comparable levels as those of cIMP (Figure 1I‐K and Figure 3A,B,D). Although cGMP is a potent vasodilating factor,^13^, ^14^ inhibition of PDE by IBMX (10^−5^ mol/L) (Figure 6A,B), PDE1 by 8‐methoxymethyl‐IBMX (4 × 10^−5^ mol/L) (Figure 6C,D) or PDE5 by zaprinast (10^−5^ mol/L) (Figure 6E,F) augmented hypoxic constriction, respectively. Consistently, treatment with milrinone (5 × 10^−6^ mol/L) (Figure 6G,H) or rolipram (5 × 10^−6^ mol/L) (Figure 6I,J) failed to duplicate the phenomenon. These results were corroborated in experiments in coronary arteries treated with a NO donor (DETA NONOate) (3 × 10^−6^ mol/L) with the inclusion of NLA (10^−4^ mol/L), in which IBMX (10^−5^ mol/L), 8‐methoxymethyl‐IBMX (4 × 10^−5^ mol/L) and zaprinast (10^−5^ mol/L) (Figure S3A‐D), but not milrinone (5 × 10^−6^ mol/L) or rolipram (5 × 10^−6^ mol/L) (Figure S3E,F) robustly potentiated NO‐mediated hypoxic responses. Taken together, these data suggest that the enhancement of NO‐dependent hypoxic vasoconstriction of coronary artery by PDE1 or PDE5 inhibition is achieved via elevating intracellular cIMP levels in smooth muscle cells.

### 3.5 Hypoxia‐induced relaxation of porcine coronary artery is independent of cIMP levels

We found that hypoxia induced a substantial relaxation response after the acute vasoconstriction in porcine coronary artery. This hypoxia‐induced relaxation was greater in coronary artery pre‐treated with nitro‐l‐arginine than in the control (Figure S4A). However, there was no significant difference of the relaxation responses in coronary arteries with or without endothelium and in endothelium‐denuded coronary arteries treated with or without cIMP (Figure S4B,C). This indicates that hypoxia‐induced relaxation after vasoconstriction in porcine coronary artery is independent of cIMP levels. Consistently, we found that PDE inhibitors had little effect on hypoxia‐induced relaxation in endothelium‐denuded coronary arteries treated with cIMP (Figure S5). Moreover, we found that IBMX and 8‐methoxymethyl‐IBMX, but not zaprinast, milrinone or rolipram, significantly abolished the relaxation response after hypoxic constriction in intact coronary artery (Figure S6). Nevertheless, there was no difference in relaxations between arteries treated with NLA or NLA plus IBMX (Figure S6A).

## 4 DISCUSSION

cIMP is a putative second messenger that mediates hypoxic constriction of porcine coronary artery under the control of the NO‐sGC singling pathway.^9^, ^20^, ^21^, ^35^, ^36^ Nevertheless, it remains unclear as to how its levels are regulated in living systems; there is solid evidence to support the ‘second messenger’ hypothesis for cIMP. Moreover, the consequences of regulation of cIMP levels on hypoxic constriction are largely unknown.

Normally, the activation of sGC triggers increasing level of cGMP to regulate vessel tension.^13^, ^14^ It has been supposed that up‐regulated cGMP levels are indispensable for hypoxic constriction.^7^ This hypothesis was challenged by another study that found that hypoxic constriction was independent of cGMP.^10^ Subsequently, we found that cIMP but not cGMP determined the hypoxic response.^9^ In the present study, exogenous supply of cGMP analogs failed to restore hypoxic response inhibited by ODQ. Nevertheless, cIMP initiated robust hypoxic constriction in endothelium‐denuded porcine coronary artery. UPLC/MS/MS analysis also revealed that cIMP levels were substantially augmented by hypoxia whereas there was no significant reduction in cGMP levels. These data suggest that cIMP, but not cGMP, is a key determinant of hypoxia‐induced vasoconstriction in porcine coronary arteries.

As in the case of cGMP, levels of cIMP were shown to be regulated by several PDEs in studies using purified human PEDs and 3′,5′‐cyclic nucleotides.^22^, ^23^ In the present study, we found that inhibition of PDE activity using a broad‐spectrum inhibitor IBMX delayed cIMP degradation and thereby increased its intracellular levels and enhanced hypoxic constriction in endothelium‐denuded porcine coronary artery treated with exogenous cIMP and in the presence of NLA. Further analysis suggested that PDE inhibitors including 8‐methoxymethyl‐IBMX (PDE 1 inhibitor) and zaprinast (PDE 5 inhibitor), but not milrinone (PDE3 inhibitor) or rolipram (PDE 4 inhibitor), were involved in this regulation. These results are consistent with those of previous studies ^22^, ^23^ suggesting that cIMP levels are regulated by PDE1 and PDE5 but not PDE4. Nevertheless, these results were inconsistent with our finding that inhibition of PDE3 had little effect on cIMP levels and related hypoxic constriction.

When pure cIMP was applied exogenously, cGMP levels were far lower than its physiological level and less than cIMP levels measured in endothelium‐denuded coronary artery in the presence of NLA (Figure 3). However, cGMP levels were comparable to those of cIMP in intact coronary arteries exposed to hypoxia (Figures 1 and 3). cGMP levels were also regulated by PDE1 and PDE5.^37^, ^38^, ^39^ In real hypoxic conditions, we found that inhibition of PDE1 and PDE5 induced significant up‐regulation of cIMP and cGMP levels in coronary arteries, as well as increased vessel tension. We also found that treatments with all PDE inhibitors induced significant reductions of vessel tension raised by U‐46619 before exposure to hypoxia, suggesting that the vasodilating effects of cGMP (by the inhibitions of PDE1 and PDE5^37^, ^38^, ^39^) and cAMP (by the inhibitions of PDE3 and PDE4^37^, ^40^, ^41^) are augmented (Figure 6). In the present study, the amplitude of hypoxic constriction is independent of the degree of vasocontraction induced by a certain range of U‐46619 concentration (Figure S7A,B). If the decreased vessel tension remains above basal levels (29.1 ± 6.6‐47.3 ± 11.2 per cent of the contraction induced by U‐46619 was retained; Figure S7C), inhibition of PDE1 and/or PDE5 by IBMX, 8‐methoxymethyl‐IBMX or zaprinast elicited stronger hypoxic constriction in coronary arteries than did pure hypoxic treatment (Figure 6). Although the vessel tension was reduced (50.1 ± 3.7‐59.5 ± 8.9 per cent of the contraction induced by U‐46619 was retained; Figure S7C), hypoxia nevertheless induced marked constriction in the presence of milrinone or rolipram, which was not greater than that of control (Figure 6). However, if vessel tension is reduced to basal levels or even lower, these hypoxic responses were completely compromised (Figure S8). These findings suggest that, in physiological situations, intracellular levels of cGMP are up‐regulated by PDE1 or PDE5 inhibition, as is the case of cIMP, and that cAMP levels are up‐regulated by PDE3 or PDE4 inhibition; these three factors have opposite effects on vessel tension. Whether there is a hypoxic constriction or its enhancement is determined by two forces: one is the vasoconstricting effect of cIMP and the other is the vasodilating effect of cGMP and/or cAMP.^20^ When the inhibition of PDE is maintained at certain level with retained vessel tension and the vasoconstricting force of cIMP is stronger than the vasodilating force of cGMP and/or cAMP, hypoxic constriction or its amplification occurs in coronary arteries. Otherwise, the even greater inhibition of PDE, which causes vessel tension equal to or lower than the base level, leads to sustained reduction of vessel tension.

After hypoxia induced an acute constriction in porcine coronary artery, there was a substantial relaxation response that was also a vital factor determining the vessel tension of coronary artery. We found that there was no significant difference of the hypoxia‐induced relaxation responses in coronary arteries with or without endothelium and in endothelium‐denuded coronary arteries treated with or without cIMP. Moreover, PDE inhibitors had little effect on hypoxia‐induced relaxations of endothelium‐denuded coronary arteries treated with cIMP. Taken together, these data suggest that hypoxia‐induced relaxation after vasoconstriction in porcine coronary artery is independent of cIMP levels.

In addition, we found that the hypoxia‐induced relaxation was greater in arteries pre‐treated with nitric oxide synthase inhibitor, nitro‐l‐arginine, compared to the control. We speculate that one reason that may influence this relaxation response is due to the amplitude and duration of hypoxia‐induced constriction. The greater amplitude and longer duration of the constricting response remarkably delays the time to dilate coronary artery. Furthermore, the difference of hypoxia‐induced relaxation between coronary artery treated with NLA and endothelium‐denuded coronary artery indicates that there may be some endothelium‐derived relaxing factors released by hypoxia.^42^ We also found that IBMX and 8‐methoxymethyl‐IBMX significantly abolished relaxations after hypoxic contraction, which may be due to greater amplitudes and longer durations of constricting responses induced by these PDE inhibitors. Nevertheless, there was no difference between coronary arteries treated with NLA or NLA plus IBMX, suggesting that PDE inhibitors may not affect the release of endothelium‐derived relaxing factors. In this situation, it is more likely that IBMX and 8‐methoxymethyl‐IBMX delayed hypoxia‐induced relaxation responses by inducing stronger contractions than those of controls.

Taken together, our findings suggest that intracellular cIMP is probably regulated by PDE1 and PDE5‐solid evidence to further support the notion that cIMP may function as a second messenger. The inhibition of PDE1 or PDE5 substantially elevates cIMP levels and augments related hypoxic constriction in endothelium‐denuded porcine coronary artery supplemented with exogenous cIMP and in the presence of NLA. In intact coronary artery, inhibition of PDE1 or PDE5 at certain levels increases both cIMP and cGMP levels, but still potentiates the hypoxic response. However, when PDE is inhibited by a greater dose of its inhibitor, the hypoxic response is largely abrogated. Our findings provide a novel mechanistic understanding of the regulation of cIMP within living tissue (coronary artery). Considering that the hypoxic constriction may lead to CAS and cardiac dysfunction and that PDE1 and PDE5 inhibitors are therapies to treat various disease,^43^, ^44^, ^45^, ^46^, ^47^ our data may also provide clinical references with the use of these inhibitors in patients with coronary artery disease exposed to repeated hypoxia (as in the case of sleep apnoea^4^, ^5^, ^6^).

## CONFLICT OF INTEREST

The authors confirm that there are no conflicts of interest.

## AUTHOR CONTRIBUTIONS

**Yan Nan:** Conceptualization (supporting); data curation (supporting); formal analysis (equal); funding acquisition (supporting); methodology (equal); resources (supporting); validation (equal); visualization (equal); writing‐original draft (equal); writing‐review and editing (equal). **Xueqin Zeng:** Data curation (supporting); formal analysis (equal); methodology (supporting); validation (equal); visualization (equal); writing‐original draft (supporting). **Zhiyi Jin:** Data curation (supporting); formal analysis (equal); methodology (supporting); validation (equal); visualization (equal); writing‐original draft (supporting). **Na Li:** Formal analysis (supporting); methodology (supporting); validation (supporting); visualization (supporting). **Zhengju Chen:** Data curation (supporting); methodology (supporting); validation (supporting); visualization (supporting). **Jiantong Chen:** Methodology (supporting); validation (supporting); visualization (supporting). **Dezhong Wang:** Funding acquisition (supporting); resources (supporting); validation (supporting); visualization (supporting). **Yang Wang:** Conceptualization (equal); data curation (supporting); formal analysis (supporting); funding acquisition (equal); investigation (supporting); project administration (supporting); resources (equal); supervision (supporting); validation (equal); visualization (equal); writing‐review and editing (equal). **Zhenlang Lin:** Conceptualization (equal); data curation (supporting); formal analysis (supporting); investigation (equal); project administration (equal); resources (supporting); supervision (equal); validation (equal); visualization (equal); writing‐original draft (equal); writing‐review and editing (equal). **Lei Ying:** Conceptualization (lead); data curation (lead); formal analysis (equal); funding acquisition (lead); investigation (lead); methodology (equal); project administration (lead); resources (equal); supervision (lead); validation (lead); visualization (equal); writing‐original draft (lead); writing‐review and editing (lead).

## Supporting information

Supplementary Material

## Data Availability

The data sets used and/or analysed during the current study are available from the corresponding author on reasonable request.
