# Supplementary material for: PDE1 or PDE5 inhibition augments NO‐dependent hypoxic constriction of porcine coronary artery via elevating inosine 3′,5′‐cyclic monophosphate level
Source: J Cell Mol Med. 2020 Nov 9;24(24):14514–24. doi: 10.1111/jcmm.16078 (PMC7754025; doi:10.1111/jcmm.16078)
Supplement: Supplementary file 1 — Supplementary Material [file JCMM-24-14514-s001.docx]

**SUPPLEMENTARY INFORMATION**

**PDE1 or PDE5 inhibition augments NO-dependent hypoxic constriction of porcine coronary artery via elevating inosine 3′,5′-cyclic monophosphate level**

**
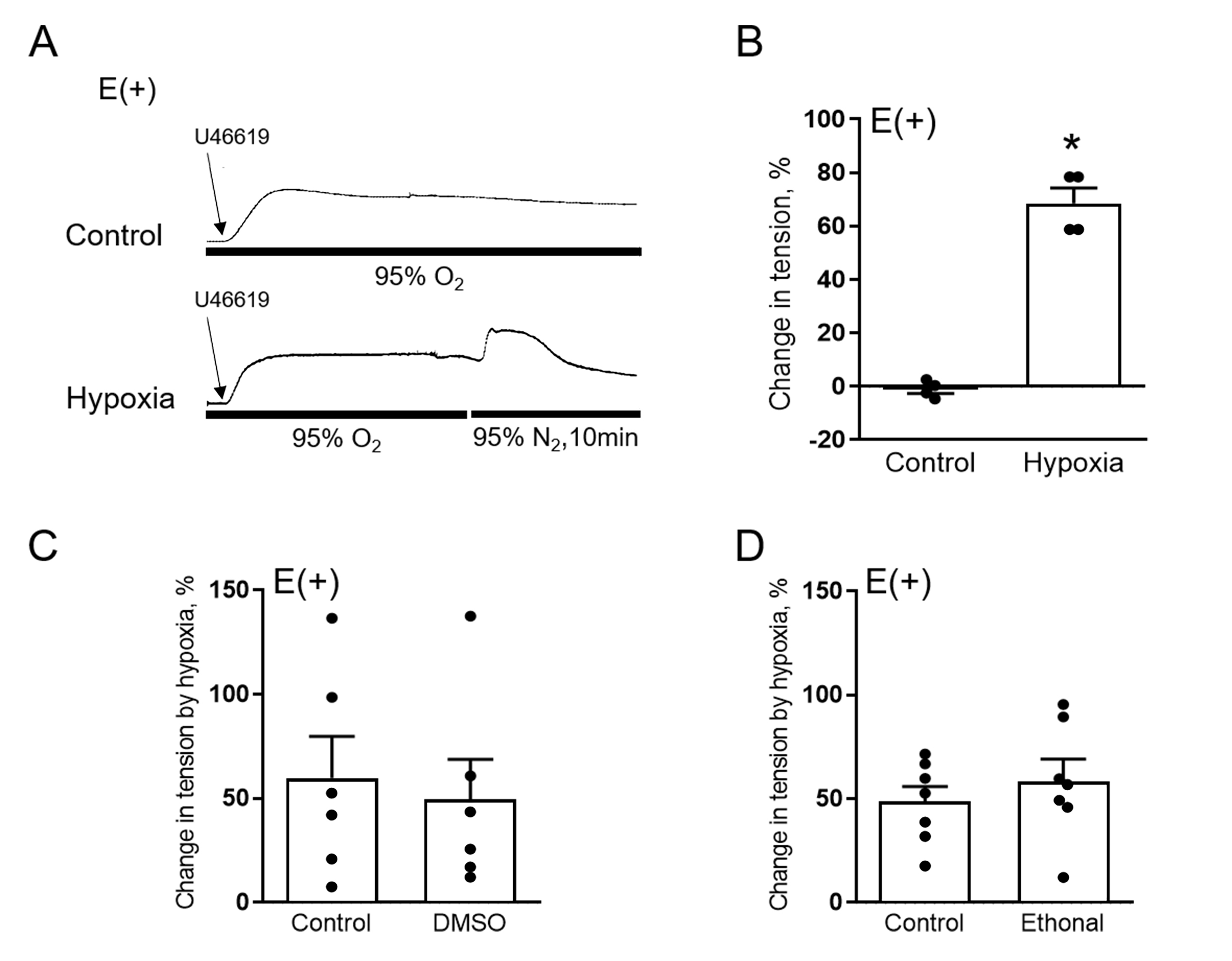
**

**Figure S1.** **Hypoxic vasoconstriction of intact porcine coronary artery is not affected by low levels of DMSO and ethanol**

**(A-D)** Original traces **(A)** and summaries **(B-D)** of hypoxic responses of porcine coronary arteries pretreated with indomethacin (10^-5^ M) for at least 30 mins, contracted with U-46619 (3 x10^-7^ M) and incubated with DMSO (final concentration, 0.01%), ethanol (final concentration, 0.01%) or solvent control (n=4-7). E (+), with endothelium. All data are presented as mean ± SEM. *p<0.05 vs control. Statistical comparisons in B-D are unpaired two-tailed Student’s t tests.

**
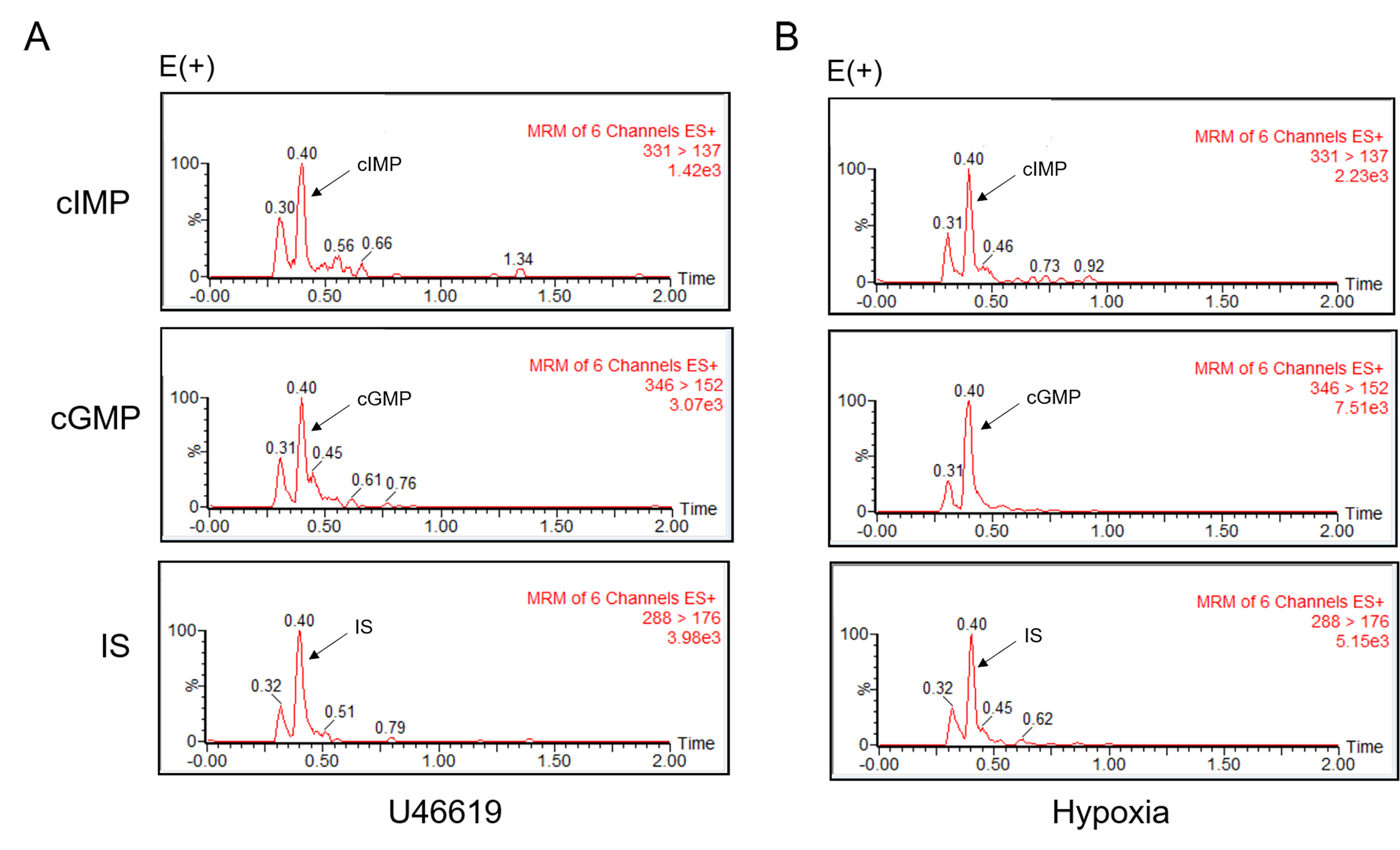
Figure S2. Original ultra-performance liquid chromatography-MS/MS chromatograph with retention time**

**(A and B)** Original ultra-performance liquid chromatography-MS/MS chromatograph of cIMP, cGMP and IS in porcine coronary arteries pretreated with indomethacin (10^-5^ M) for at least 30 mins, incubated with U-46619 (3 x 10^-7^ M) for 30 mins **(A)** and exposed to hypoxia for 3 mins **(B)**. Tenofovir (10 ng/ml) was served as an internal standard (IS).

**
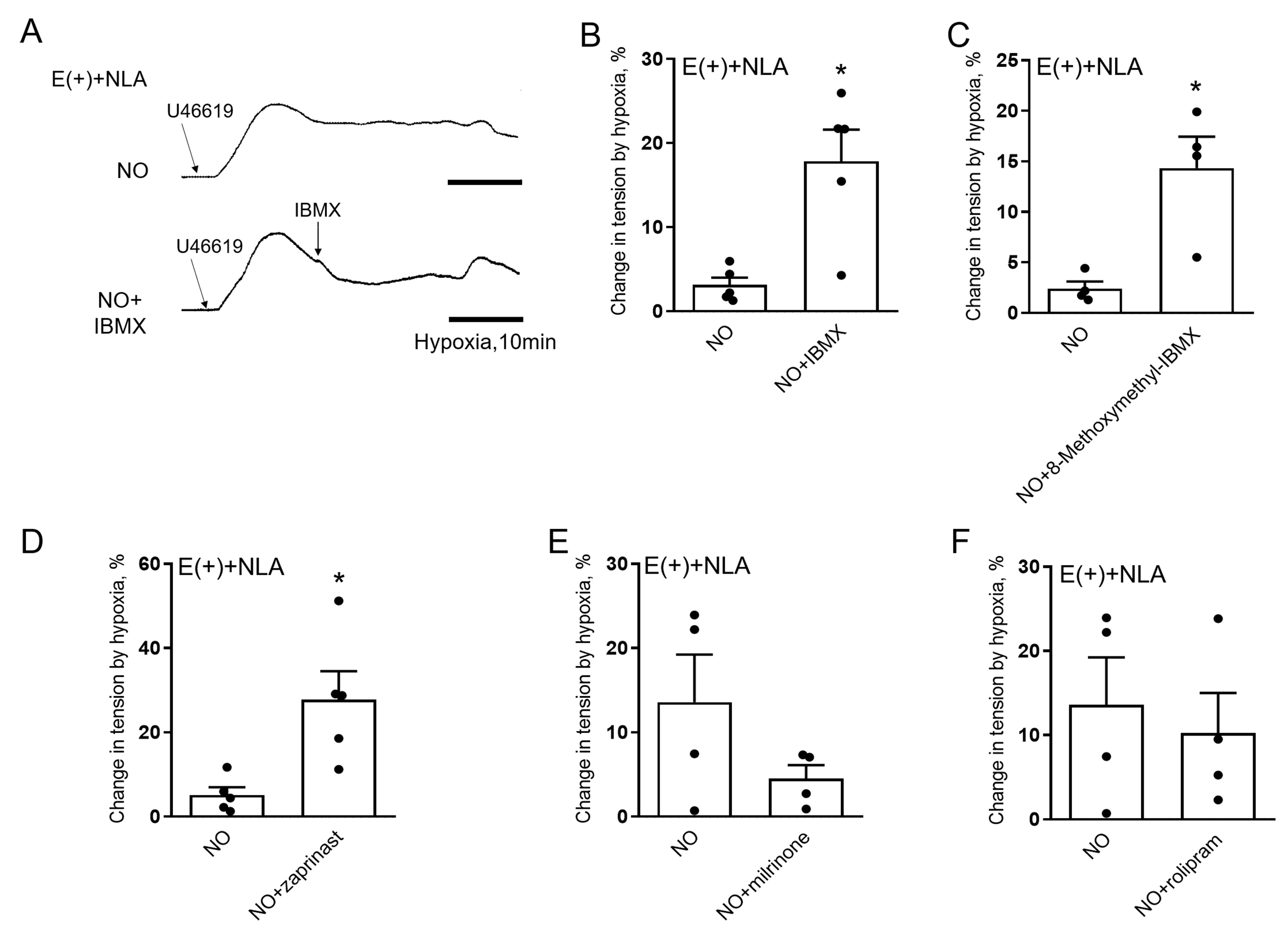
Figure S3. NO-mediated hypoxic vasoconstriction of porcine coronary artery is augmented by the inhibition of PDE1 or PDE5**

**(A-F)** Original traces **(A)** and summaries **(B-F)** of hypoxic responses of coronary arteries pretreated with indomethacin (10^-5^ M), NLA (10^−4^ M) and NO (3 x 10^−6^ M) for at least 30 mins, contracted with U-46619 (3 x10^-7^ M) and incubated with IBMX (10^-5^ M) **(A, B)**, 8-Methoxymethyl-IBMX (4 x10^-5^ M) **(C)**, zaprinast (10^-5^ M) **(D)**, milrinone (5 x 10^-6^ M) **(E)**, rolipram (5 x 10^-6^ M) **(F)** or solvent control (n=4-5). E (+), with endothelium. All data are presented as mean ± SEM. *p<0.05 vs NO. Statistical comparisons in B-F are unpaired two-tailed Student’s t tests.

**
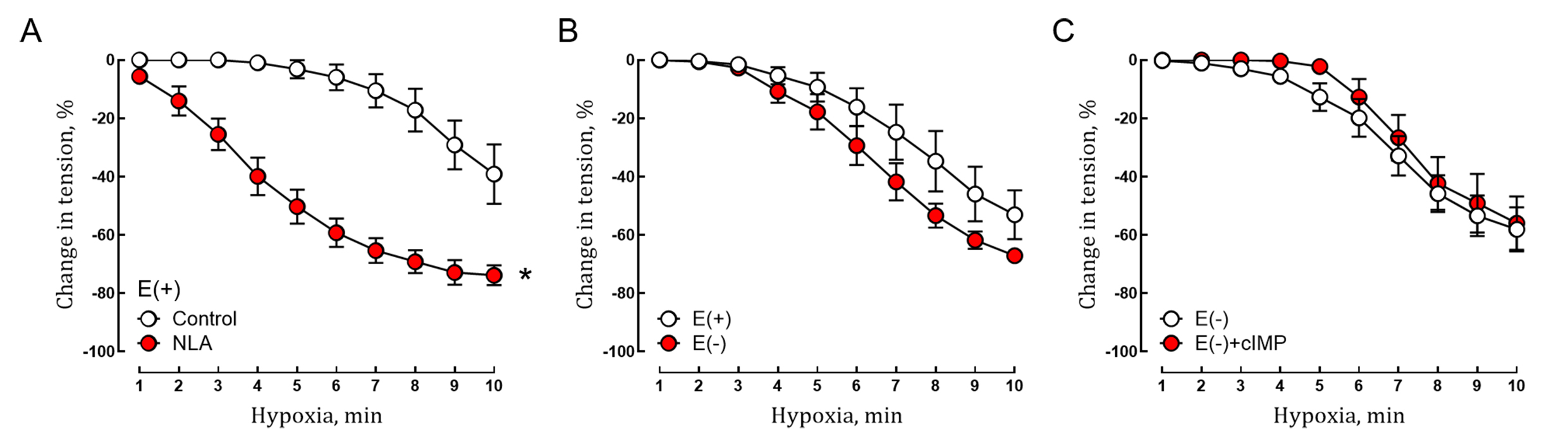
**

**Figure S4. Hypoxia-induced relaxation of intact porcine coronary artery is enhanced by the inhibition of nitric oxide synthase**

(A) Summaries of hypoxic relaxations of porcine coronary arteries pretreated with indomethacin (10^-5^ M) plus NLA (10^−4^ M) or solvent control for at least 30 mins and contracted with U-46619 (3 x 10^-7^ M) (n=8). (B) Summaries of hypoxic relaxations of porcine coronary arteries (with or without endothelium) pretreated with indomethacin (10^-5^ M) for at least 30 mins and contracted with U-46619 (3 x 10^-7^ M) (n=7). (C) Summaries of hypoxic relaxations of endothelium-denuded porcine coronary arteries pretreated with indomethacin (10^-5^ M) for at least 30 mins, contracted with U-46619 (3 x 10^-7^ M) and incubated with cIMP (10^-4^ M) (n=9). E (+), with endothelium. E (-), without endothelium. All data are presented as mean ± SEM. *p<0.05 vs Control. Statistical comparisons in A-C are two-way ANOVA tests with Sidak's multiple comparisons tests.

**
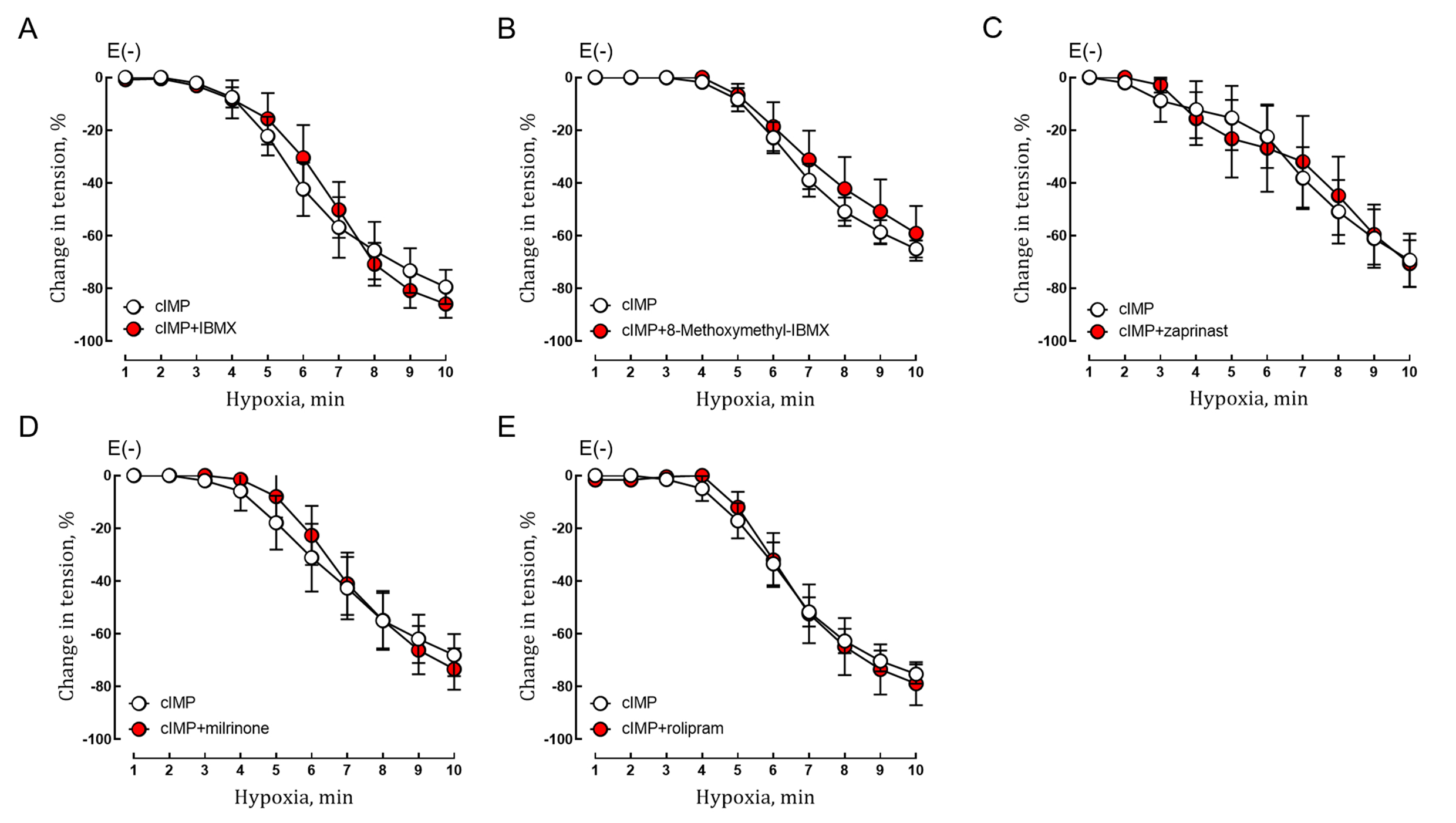
**

**Figure S5. Hypoxia-induced relaxation of endothelium-denuded porcine coronary artery is not affected by inhibitions of PDEs**

(A-E) Summaries of hypoxic relaxations of endothelium-denuded coronary arteries pretreated with indomethacin (10^-5^ M) and NLA (10^-4^ M) for at least 30 mins, contracted with U-46619 (6 x 10^-8^ M) and incubated with exogenous cIMP (10^-4^ M) plus IBMX (3 x 10^-5^ M) (A), 8-methoxymethyl-IBMX (4 x 10^-5^ M) (B), zaprinast (10^-5^ M) (C), milrinone (5 x 10^-6^ M) (D), rolipram (5 x 10^-6^ M) (E) or solvent control (n=6-7). E (-), without endothelium. All data are presented as mean ± SEM. Statistical comparisons in A-E are two-way ANOVA tests with Sidak's multiple comparisons tests.

**
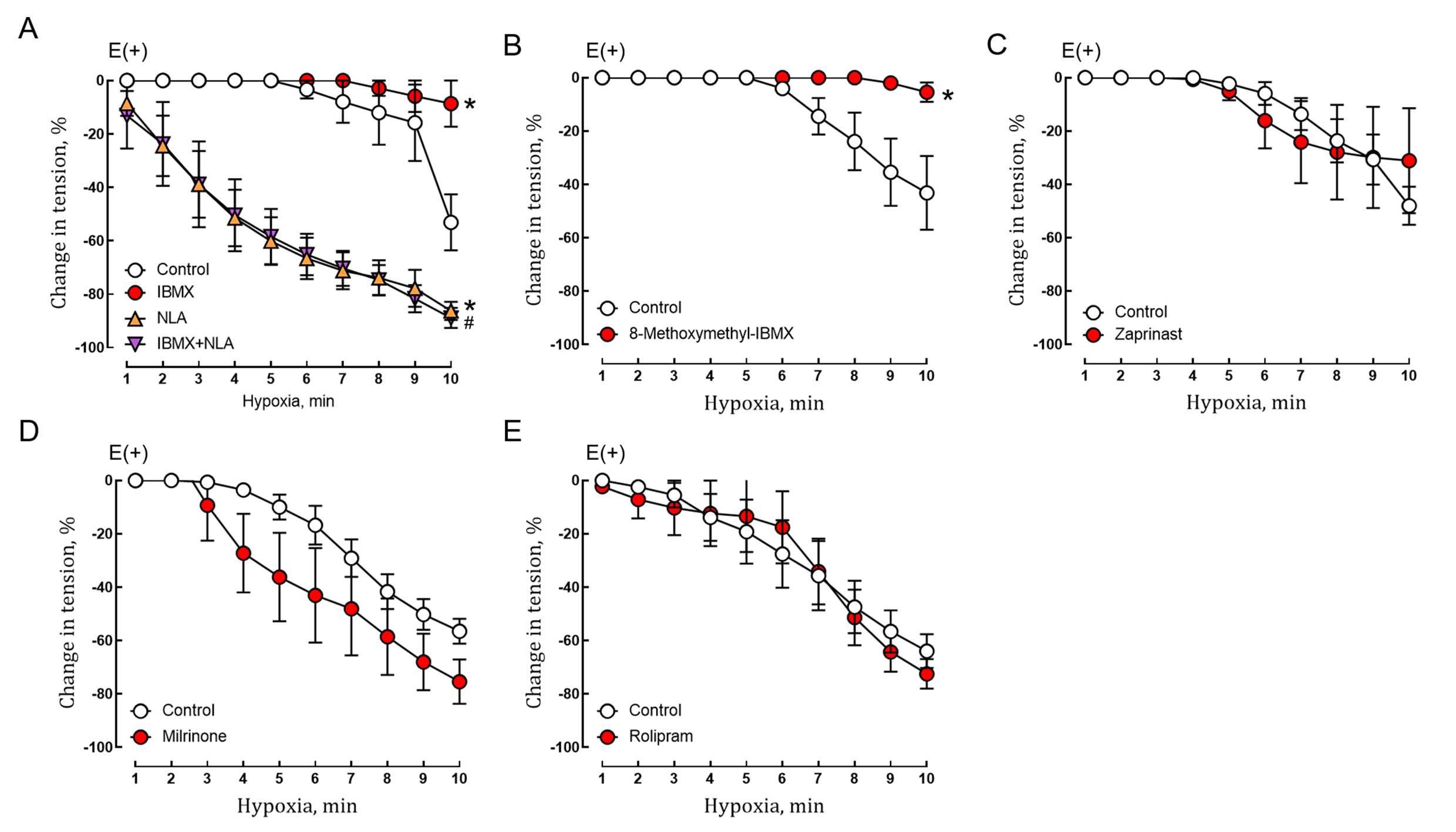
**

**Figure S6. Hypoxia-induced relaxation of intact porcine coronary artery is inhibited by IBMX and 8-methoxymethyl-IBMX**

(A-E) Summaries of hypoxic relaxations of intact coronary arteries pretreated with indomethacin (10^-5^ M) and/or NLA (10^-4^ M) for at least 30 mins, contracted with U-46619 (3 x10^-7^ M) and incubated with IBMX (10^-5^ M) (A), 8-methoxymethyl-IBMX (4 x 10^-5^ M) (B), zaprinast (10^-5^ M) (C), milrinone (5 x 10^-6^ M) (D), rolipram (5 x 10^-6^ M) (E) or solvent control (n=5-6). E (+), with endothelium. All data are presented as mean ± SEM. *p<0.05 vs control; ^#^p<0.05 vs IBMX. Statistical comparisons in A-E are two-way ANOVA tests with Sidak's multiple comparisons tests.

**
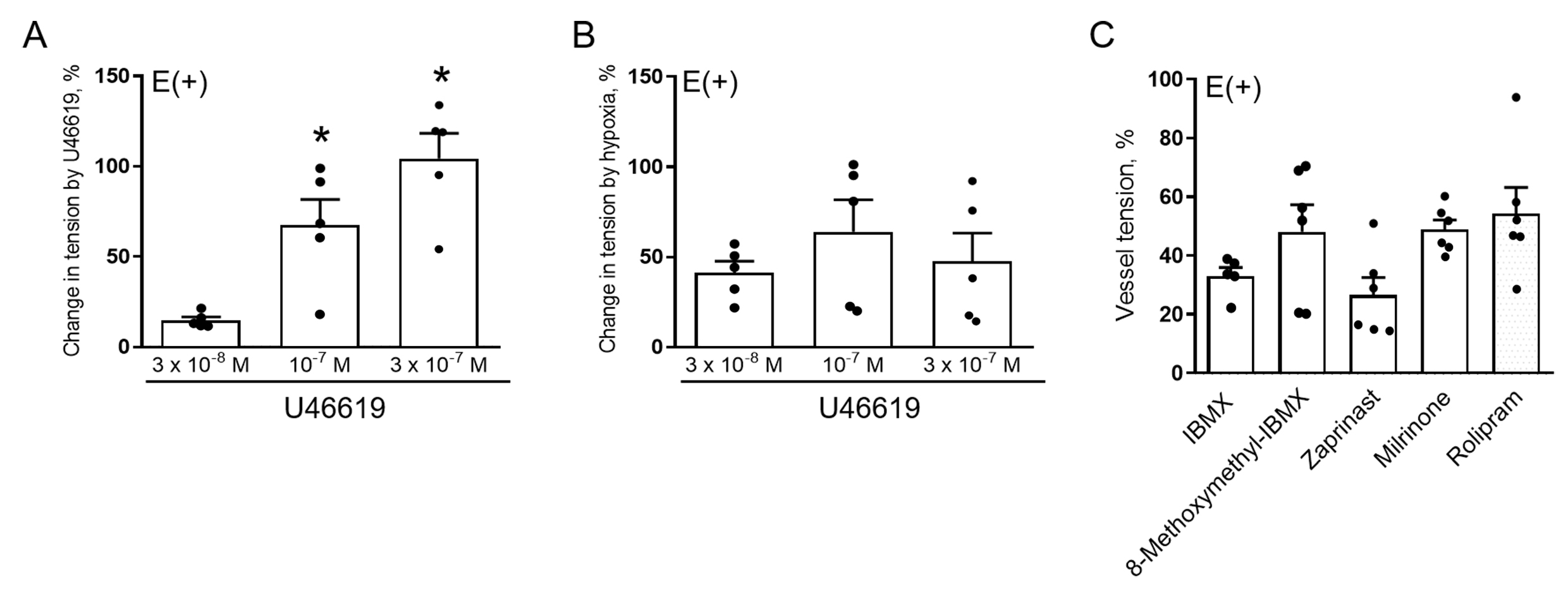
Figure S7. The amplitude of hypoxic constriction in intact porcine coronary artery is independent of the degree of vasocontraction induced by a certain range of U-46619 concentration**

**(A)** Summaries of contractions in porcine coronary artery pretreated with indomethacin (10^-5^ M) for at least 30 mins and contracted with U-46619 (3 x10^-8^, 10^-7^, or 3 x10^-7^ M) (n=5). E (+), with endothelium. All data are presented as mean ± SEM. U-46619-induced contractions were expressed as percentage of the reference contraction to the second KCl treatment (100 mM) (taken as 100%).

**(B)** Summaries of hypoxic responses of porcine coronary arteries pretreated with indomethacin (10^-5^ M) for at least 30 mins and contracted with U-46619 (3 x10^-8^, 10^-7^, or 3 x10^-7^ M) (n=5). E (+), with endothelium. All data are presented as mean ± SEM. Hypoxia-induced contractions were expressed as percentage of the reference contraction to the second KCl treatment (100 mM) (taken as 100%).

**(C)** Summaries of vessel tensions of intact coronary arteries pretreated with indomethacin (10^-5^ M) for at least 30 mins, contracted with U-46619 (3 x10^-7^ M) and incubated with IBMX (10^-5^ M), 8-Methoxymethyl-IBMX (4 x 10^-5^ M), zaprinast (10^-5^ M), milrinone (5 x 10^-6^ M), rolipram (5 x 10^-6^ M) (n=5-6). E (+), with endothelium. All data are presented as mean ± SEM.

*p<0.05 vs 3 x 10^-8^ M. Statistical comparisons in A-C are one-way ANOVA tests with Tukey's multiple comparisons tests.

**
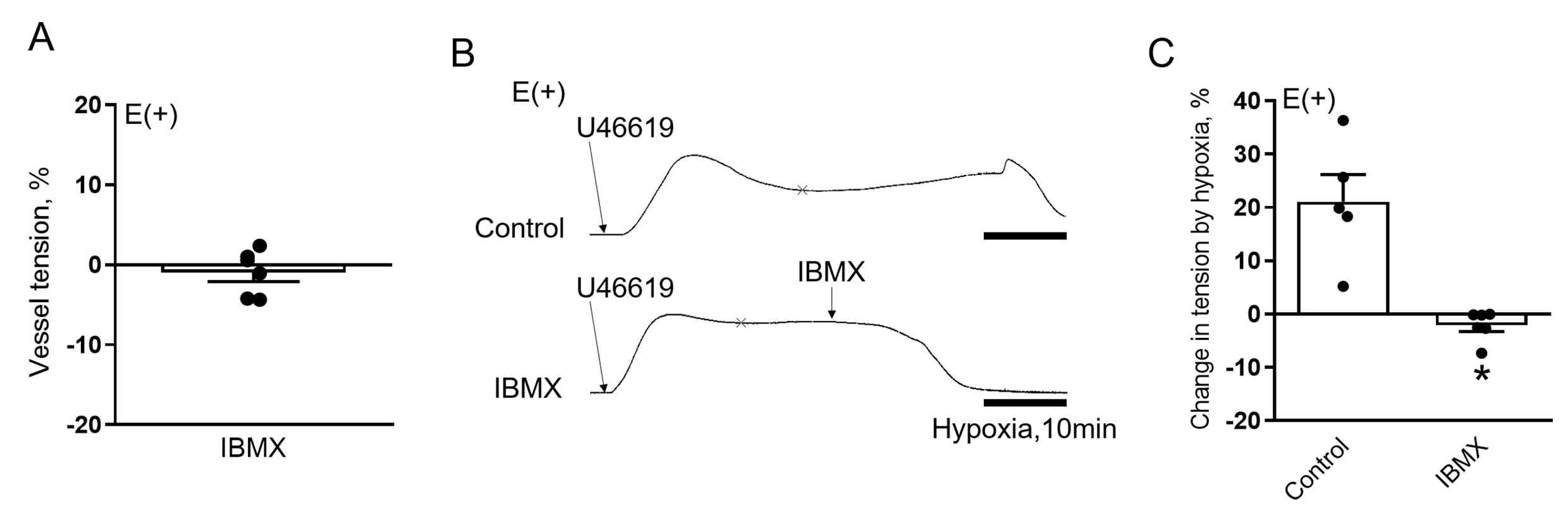
Figure S8. Hypoxic vasoconstriction of intact porcine coronary artery is inhibited by higher levels of IBMX**

**(A)** Summaries of vessel tensions of intact coronary arteries pretreated with indomethacin (10^-5^ M) for at least 30 mins, contracted with U-46619 (3 x10^-7^ M) and incubated with IBMX (10^-4^ M) (n=6). E (+), with endothelium. All data are presented as mean ± SEM.

**(B and C)** Original traces **(B)** and summaries **(C)** of hypoxic responses of porcine coronary arteries pretreated with indomethacin (10^-5^ M) for at least 30 mins, contracted with U-46619 (3 x10^-7^ M) and incubated with IBMX (10^-4^ M) or solvent control (n=5-6). E (+), with endothelium. All data are presented as mean ± SEM. *p<0.05 vs control. Statistical comparison in C is unpaired two-tailed Student’s t test.
